# Accuracy of optical coherence tomography (OCT) in pachymetry for glaucoma patients

**DOI:** 10.1186/s12886-015-0116-x

**Published:** 2015-09-29

**Authors:** Marcelo Ayala, Robert Strandås

**Affiliations:** Eye Department, Skaraborgs Hospital, Skövde, Sahlgrenska Academy, Gothenburg University & Karolinska Institute, 541 85 Skövde, Sweden; Eye Department, Skaraborgs Hospital, Skövde, Sweden

**Keywords:** Accuracy, Optical coherence tomography, Ultrasound pachymetry, Pachymetry

## Abstract

**Background:**

Central corneal thickness (CCT) measurement has become an important test in the diagnosis and management of glaucoma. Currently, ultrasound corneal thickness measurement (pachymetry) is the most frequently used clinical technique and the gold standard to assess CCT. Newer instruments are currently available including the optical coherence tomography (OCT) instrument. The aim of the present study was therefore to evaluate the accuracy of the CCT measurements performed by three different observers, both with the OCT and ultrasound pachymetry (USP), in patients suffering from glaucoma.

**Methods:**

Patients who had been previously diagnosed with glaucoma participated in this cross-sectional study. Glaucoma was defined as patients who had at least two repeatable Humphrey visual fields showing glaucoma damage using the software 24–2, and with the optic nerve showing typical glaucoma damage. The patients CCTs were measured with OCT and USP by three different examiners.

**Results:**

Seventy eyes of 35 patients were included. The average age was 74 ± standard deviation (SD) 10.88, the average pachymetry value with OCT was 536 ± 29 μm, and the average pachymetry with USP was 532 ± 32 μm. The differences between OCT and USP were not significant (*t*-test, *p* = 0.32). The intraclass correlation coefficients were, for OCT, 0.99 [confidence interval (CI): 0.98–0.996], and for USP, 0.97 (CI: 0.95–0.98).

**Conclusions:**

Agreement among the three observers using OCT or USP for pachymetry measurements was good. OCT might be used as an alternative method for pachymetry in glaucoma patients.

## Background

Central corneal thickness (CCT) measurement has become an important test in the diagnosis and management of glaucoma. Most studies have found that increased or decreased CCT could lead to overestimating or underestimating the true intraocular pressure (IOP). Moreover, CCT is the most predictive factor for progression of ocular hypertension to glaucoma. According to the results of the Ocular Hypertension Treatment Study, an individual with a CCT measuring 40 μm thinner than the average has a 71 % greater risk of developing glaucoma [1].

Currently, ultrasound corneal thickness measurement (pachymetry) is the most frequently used clinical technique and the gold standard to assess CCT [2]. However, ultrasound pachymetry (USP) has several possible sources of error. Its accuracy depends on the placement of the probe on the cornea, and the perpendicularity of the probe in relation to the cornea is often difficult to ascertain. Before USP measurements, topical anesthesia must be instilled and this could induce bias in the measurements. Moreover, this instrument is a contact type pachymeter, requiring aseptic precautions and anesthetizing the cornea, and the further possibility of injury to the cornea [3].

Newer instruments are currently available that have the advantage of being the non-contact type. One type is the optical coherence tomography (OCT) instrument. OCT is now widely used at ophthalmology departments, mostly for measuring thickness in the retina; but the OCT instrument can be used for measuring thicknesses in the cornea and the nerve fiber layer. However, it is not known how repeatable and stable the measurements are.

Previous studies were performed in normal eyes [4–6] or eyes suffering from keratoconus [7–9]. The only confirming study regarding CCT measurements with OCT in glaucomatous eyes was that of Garcia-Median et al. [10]. The aim of the present study was therefore to evaluate the accuracy of the CCT measurements performed by three different observers using both OCT and USP in patients diagnosed with glaucoma.

## Methods

### Subjects

This cross-sectional study was composed of 35 patients (70 eyes) who had been previously diagnosed with glaucoma. Ethics approval was received from the institutional review board (Ethical approval: 717–13, Gothenburg, Sweden). The study followed the tenets of the Declaration of Helsinki. Before enrolling patients in the study, written informed consent was obtained. Recruitment was performed at the Eye Department, Skaraborgs Hospital (SkaS), Skövde, Sweden.

### Study protocol

A comprehensive medical and ocular history was obtained. Ophthalmological examination was performed before including patients in the study. Visual acuity, IOP measurements, optic nerve status, gonioscopy, Humphrey visual fields (HFA, 24–2), and presence or absence of exfoliation were registered. Visual acuity was recorded using a Snellen chart. IOP was measured using a Goldmann applanation tonometer. Three measurements were taken, and the average was calculated.

The pupils were dilated, exfoliation was checked, and exfoliation was registered as present or absent. Afterwards, the optic nerve status was evaluated using a 90-D lens and stereo photographs were taken. Previous eye surgery was also registered. Glaucoma was defined following the European Guidelines for Glaucoma, as patients who had at least two repeatable Humphrey visual fields showing glaucoma damage using the software 24–2, and with the optic nerve showing typical glaucoma damage [11].

After the examinations, patients that were to be included in the study were measured for CCT with the OCT (3D OCT-20000; Topcon Corporation, 75–1 Hasunuma-Cho, Tokyo, Japan) by three different examiners: an ophthalmologist senior consultant (MA), a resident (RS), and an ophthalmic nurse (ON). CCT measurements were also taken with USP (Tomey Pachymetry; Tomey Corp, Nagoya 451–0051, Japan) by the same examiners. The order of measurements was OCT/USP or USP/OCT, and the order of the observers who performed the measurements was chosen at random.

Each participant was positioned on the OCT headrest and requested to direct his or her gaze at the internal fixation point. The subject’s pupil was used to center the scan. Images were taken using the anterior segment option that provided a radial scan with 12 spaced lines around the central cornea. Three different examiners (MA, RS, and ON) performed the measurements, and the order of the measurements was decided at random. Only images of good quality were recorded (>60 signal strength).

CCT measurements using the USP instrument were taken by the same three examiners (MA, RS, and ON) after instillation of topical local anesthetic (0.5 % proxymetacaine hydrochloride). The order of the measurements was decided at random. The probe was directed perpendicular to the central cornea surface, three readings were taken, and average values were calculated. The USP instrument showed the CCT measurements on the display. For all patients, the times between the OCT and USP measurements were from 30 to 45 min.

### Statistics

Descriptive statistics were calculated for OCT and USP CCT measurements using SPSS version 20 (SPSS, Chicago, IL, USA). To measure differences in the CCT values between OCT and USP, a paired *t*-test was performed. Significance level was *p* < 0.05. To test agreement between the two different instruments, a Bland–Altman plot was performed. Mean differences and limits of agreement (LOA) were calculated. To estimate repeatability among the three different observers, the intraclass correlation coefficient (ICC) was calculated. The ICC test is a good option when testing quantitative measurements made by different observers measuring the same parameter. The ICC was calculated using the single measurements one-way random effects model. The ICC ranged from a value of 0 to 1, with 0 indicating no agreement, and 1 indicating absolute agreement between repeated measurements. Regarding sample size, similar studies have included around 30–40 eyes [6, 12].

## Results

### Demography

In total, 35 patients (70 eyes) were included in the study. The mean age of all patients was 74 ± 10.88 years, and the age range was 39–93 years of age. Regarding gender distribution, there were 17 male and 18 female patients included in the study. All included patients were on medical treatment with an average of 1.11 ± 1.13 active medication substances. The visual field damage was estimated using the visual field index (VFI), and was VFI = 79.93 % ± 21.23 %.

Regarding diagnostic distribution, 60 eyes suffered from primary open-angle glaucoma, and 10 eyes had exfoliation glaucoma. The included eyes were 25 eyes with pseudophakia and 45 eyes were phakic (no cataract surgery). None of the included patients underwent refractive surgery before inclusion.

### Endpoints

**Fig. 1:**
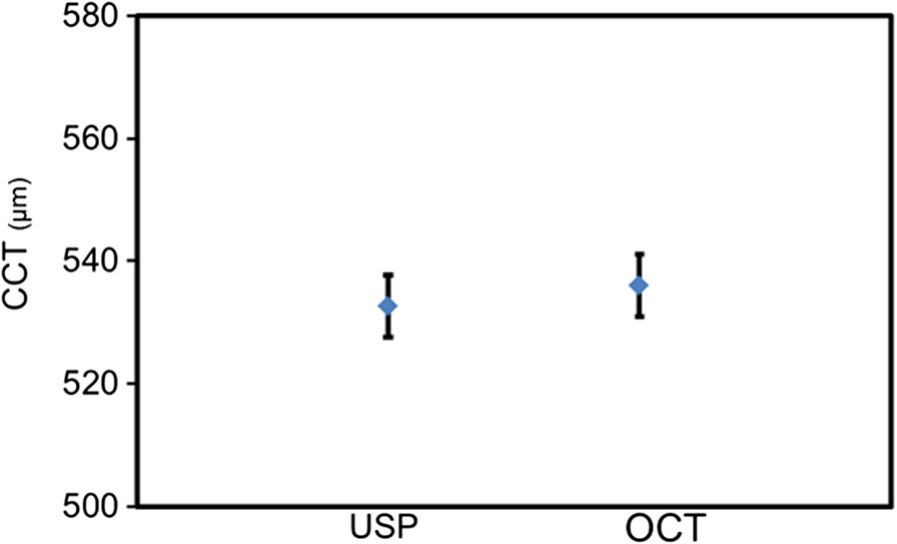
Corneal thickness measurements. Left = USP, right = OCT measurements. No significant difference was found (*t*-test; *p* = 0.32). The bars represent 95 % confidence interval (CI) for the mean

The CCT measured using OCT (536 ± 29 μm) was thicker than when measured using USP (532 ± 30 μm). However, the difference between measurements was not significant (*t*-test, *p* = 0.32) (Fig. 1).

**Fig. 2:**
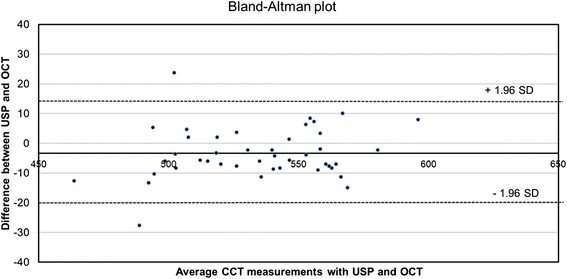
Bland–Altman plot showing the comparison between the two different instruments. Only two measurements were outside the limits of agreement (LOA)

The Bland–Altman plot revealed mean differences of −3, or 39 μm between OCT and USP. The 95 % LOA were calculated based on a 1.96 SD difference between OCT and USP. LOA was −20 μm to +13.22 μm. Only two values were situated outside the LOA: −27.6 μm and 23.6 μm (Fig. 2). Consistency among the three observers (MA, RO, and NO) was tested using the ICC test. The results for the measurements with the USP instrument were ICC = 0.97 (CI = 0.95–0.98), and were ICC = 0.99 (CI = 0.98–0.996) using the OCT instrument. The ICC result was as follows: 0.7–0.79 = good; 8–0.89 = very good; and, 0.9–0.99 = excellent consistency.

## Discussion

The main focus of our study was to determine if OCT could be a possible substitute for USP in the measurements of CCT in glaucoma patients, because OCT has the advantages of being aseptic and without the risk of contact corneal trauma. A further advantage of OCT is that it is possible to examine the results at a later time in the absence of the patient. The intra- and inter-repeatability of OCT pachymetry has also been shown to be good and perhaps even better than USP in the study by Lin et al. [13]. The findings of our study are in agreement with the studies of Garcia-Medina et al. [10] on glaucomatous eyes, in that there was no significant difference between OCT and USP when measuring CCT. Even though studies comparing OCT and USP have been reported previously [10], our study is the only one that has compared differences among different examiners.

In our study, OCT measurements overestimated CCT, when compared with USP. To our knowledge, this finding is not consistent with most other studies, including those of Garcia-Medina et al. [10], Dutta et al. [7], and Doughty [14], all describing results with OCT underestimating CCT, when compared with USP. Differences in the studies could be due to several factors, including a difference in calibration and methods of measurements. Inter-instrument variations have been demonstrated in other studies. For example, Wells et al. [15] reported a difference of up to 30 μm in CCT using different instruments. Another possible explanation for the disagreement related to previous studies could be that the present study included glaucoma patients, while other studies were based on healthy subjects. As explained by Garcia-Medina et al. [10], glaucoma is a disease that might change the characteristics of the cornea. This could explain some of the differences in CCT as measured with OCT versus USP.

The design of the study was constructed to detect if there would be any significant difference in the measured results between different examiners. The examiners ranged from a senior consultant ophthalmologist with more than 20 years of experience (MA), to a resident who had never used a USP instrument (RS). However, measuring CCT by OCT was a relatively new experience for our group. It is worth mentioning that all measurements were performed independently. We did not discuss how to measure CCT with OCT, apart from the basic technical aspects. OCT seems to be a reliable and easy way to determine CCT measurements, even when the instrument is used by someone with little prior experience.

Topical anesthesia needed for USP may cause the cornea to swell and can affect the measurements. The physical pressure from the USP on the cornea is also a factor to consider. A study by Mukhopadhyay et al. [16] showed that USP together with topical anesthesia could give variations in CCT from −10 μm to +30 μm. By randomizing OCT/USP measurements and the order of the examiners, we tried to minimize any bias from patient-examiner contact, and the effect of repeated measurements within a short time.

The study had several limitations, including the time of the day the measurements were taken. The diurnal variations with swelling of the cornea at night should be addressed. In the study by Fogagnolo et al. [17] on glaucomatous patients, mean CCT was 534 (SD = 39) μm (range, 443–637 μm) with circadian fluctuations of 16.5 (SD = 6.2) μm (range, 6–31 μm). Also, in the study by du Toit [18], the mean corneal swelling upon eye opening was 2.9 ± 0.3 % from baseline, but there was considerable individual variation, ranging from 1.3 to 7.2 %. Deswelling occurred 2 h after eye opening. For our patients, the mean time of measurement was at 10 a.m., when the cornea is thought to have recovered its natural thickness. Only four of our 37 patients were measured for CCT later than 1:00 p.m. Thus, the times between measurements might also be considered. In our study, the measurements on each patient were all done within 45 min from the first measurement to the last. During this time, the patients received topical anesthetics at some point, but this time varied from patient to patient.

There is a possibility that topical antiglaucoma medications could have affected the measurements. Medications with active substances or those with preservatives can alter corneal thickness. Regarding OCT and USP, the most important factors would be changes in corneal characteristics, including the reflection and the propagation velocity of the tissue. This is because both techniques use the time-of-flight principle, meaning the delay of a wave signal as it travels through ocular tissue, and the resulting reflected signal.

Another limitation may be the variations in measurements due to patient cooperation. The patient should be able to focus his or her gaze on something during the examination with OCT and USP. This was difficult only for a very few patients because of inability to understand instructions owing to dementia or very poor hearing. However, the same difficulties would be present for both measurement methods.

The placement of the USP probe and the OCT scans differ in procedure. The USP performs measurements with a stationary probe, while the OCT performs 12 different scans in the 12 o’clock position, and then performs an automatically averaged measurement. The observer attempts to place the OCT scan in the middle of the cornea; however, because an average measurement is calculated, the placement may not be as important as with USP.

## Conclusions

OCT and USP both showed high accuracy in pachymetry measurements even when the measurements were done by three different observers with different skills, independent of each other. OCT may therefore be a good method to measure CCT in glaucoma patients.
